# An Oral *Salmonella*-Based Vaccine Expressing Viral M43 Protein Elicits Effective Immunity Against Murine Cytomegalovirus in Mice

**DOI:** 10.3390/pathogens14090902

**Published:** 2025-09-08

**Authors:** Yujun Liu, Hao Gong, Jiaming Zhu, Fenyong Liu

**Affiliations:** 1 School of Public Health, University of California, Berkeley, CA 94720, USA; 2 Program in Comparative Biochemistry, University of California, Berkeley, CA 94720, USA

**Keywords:** cytomegalovirus, herpesvirus, human cytomegalovirus, immune response, murine cytomegalovirus, oral vaccine, *Salmonella*

## Abstract

Human cytomegalovirus (HCMV) is the leading viral cause of congenital infections and causes substantial morbidity and mortality in neonates and immunosuppressed people. Generating an anti-HCMV vaccine is required for preventing viral-associated diseases and infections. Oral vaccines based on attenuated *Salmonella* are an attractive solution, since these vaccines can be applied orally and easily for mass immunization. In this report, we constructed an attenuated *Salmonella* strain for the expression of the murine cytomegalovirus (MCMV) M43 protein and studied its ability as an oral vaccine candidate to stimulate antiviral immunity in mice. In orally immunized mice, the constructed vaccine, Sal-M43, elicited both serum IgG and mucosal IgA levels as well as T cell responses that were specific against the MCMV M43 protein. Moreover, the Sal-M43 immunization substantially inhibited the viral growth and infection in various organs and tissues and offered complete immune protection against both intraperitoneal and intranasal MCMV challenges. Thus, the *Salmonella*-based vaccine expressing the M43 antigen is effective in inducing anti-MCMV immunity. These findings also reveal the promise of developing oral anti-CMV vaccines based on attenuated *Salmonella* vectors expressing different viral antigens.

## 1. Introduction

Human cytomegalovirus (CMV), alias HCMV, is a member of the human herpesvirus family, which also includes herpes simplex virus (HSV) 1 and 2, varicella zoster virus, Epstein–Barr virus, human herpesvirus 6 and 7, and Kaposi’s sarcoma-associated herpesvirus [1,2]. Like all herpesviruses, HCMV engages in acute lytic infection to produce virus progeny in addition to establishing life-long latent infections. Both viral lytic and latent infections play important roles in the HCMV-associated pathogenesis [1].

A medically important virus, HCMV is the leading viral cause of congenital infections [3,4] and represents one of the most common opportunistic pathogens to cause substantial morbidity and mortality in immunosuppressed people, including HIV-positive individuals and organ transplant recipients [1]. Moreover, the virus is ubiquitous, infecting more than 70% of the US population [5,6]. Thus, the HCMV infection affects a substantial portion of the global population due to its ubiquitous presence and presents a substantial economic burden to society due to its congenital infections, which usually lead to neurological disorders and neurodevelopmental sequelae [7]. Unfortunately, currently FDA-approved anti-HCMV compounds cannot eliminate viral latent infections, and no FDA-approved anti-HCMV vaccines are currently available. Thus, generating an anti-HCMV vaccine is required for the prevention of virus-associated diseases and infections [8,9].

Attenuated *Salmonella* strains are promising gene delivery vectors for gene therapy and vaccine development that may be administered orally [10,11,12,13]. Attenuated *Salmonella* mutants have been generated to carry plasmid constructs with transgene expression cassettes. When used to infect human cells, these *Salmonella*-based vectors were lysed and released plasmid DNAs inside the cells, resulting in the expression of the transgene [14,15]. Members of the type III secretion system (T3SS) of *Salmonella* pathogenicity island 2 (SPI-2) are major bacterial virulence factors that contribute significantly to *Salmonella* survival and replication in infected cells [16,17]. Attenuated *Salmonella* strains with the disruption of the expression of these genes are expected to exhibit reduced virulence and cytotoxicity, improved lysis, and increased gene transfer [14,18].

With a narrow host range, HCMV is capable of reproducing only in human cells [1,2]. Animal cytomegalovirus infections of their corresponding hosts, such as the murine cytomegalovirus (MCMV) infection of mice, are excellent models for understanding the HCMV pathogenesis and for developing an anti-CMV vaccine [19]. Scientists have studied various types of anti-CMV vaccines, including recombinant protein and DNA vaccines, virus-like particle (VLP) vaccines, and conventional inactivated whole viral antigen vaccines [8]. When vaccinated mice were challenged with an MCMV infection, some of these vaccines could elicit substantial anti-MCMV immunity [20,21,22,23,24].

In the current investigation, we generated a weakened *Salmonella* mutant, ST625, by deleting the spiR gene. The SpiR protein is a major regulator controlling the expression of many components of the T3SS of *Salmonella* SPI-2 [25,26]. A functional ST625-based vaccine, Sal-M43, was constructed to express the MCMV M43 protein as the antigen for vaccination. M43 and its HCMV homolog, UL43, are tegument proteins found in the viral infectious particles [27,28,29] and therefore are potential antigens for vaccination against CMV infections. MCMV mutants with mutations at M43 replicated well in some cell lines but were attenuated in virus production in other cell lines in vitro [30,31]. We have previously shown that an MCMV mutant with a transposon mutation at M43 is defective in growth in salivary glands in infected mice and that M43 functions to modulate the T helper cell response [31,32]. Like M43, UL43 is also dispensable for HCMV replication in vitro, as viral mutants with mutations inactivating UL43 grew well in human foreskin fibroblasts [33,34]. UL43 functions to cooperate with the HCMV UL24 protein to alter the UL16 binding protein 1 level and SAMHD1 protein cellular distribution [35,36]. However, it is currently not known if M43 or UL43 can serve as anti-CMV vaccine candidates.

We show in this report that the generated *Salmonella*-based vaccine, Sal-M43, showed remarkable attenuation in killing mice in vivo. Vaccine Sal-M43 elicited a strong anti-MCMV immunity in the orally vaccinated mice. Moreover, the Sal-M43 vaccination protected the animals from the MCMV challenge. This report presents the first direct evidence to suggest that oral vaccines based on the *Salmonella* strain ST625 can be used against CMV infections.

## 2. Materials and Methods

### 2.1. Viruses, Cells, and Constructs

We cultured mouse J774 macrophages and NIH 3T3 cells in Dulbecco’s modified Eagle medium (DMEM) supplemented with 10% NuSerum (Becton Dickenson, Mountain View, CA, USA) and propagated MCMV Smith strain (ATCC, Manassas, VA, USA) and m-M43 mutant as described previously [31,37]. We employed the BAC-mid method [38] to generate mutant m-M43 by deleting the encoding sequence of M43 open reading frame (coordinates 55354–57144) [39] and verified the M43 deletion mutation through restriction enzyme and sequencing analyses. We used M43 peptides to generate the rabbit anti-M43 antisera (Promab, Inc., Richmond, CA, USA).

We created the M43 expression construct pEXP-M43 by inserting the DNA for the M43 encoding sequence into expression vector pEXP-VAX, which was derived from construct pVAX (Invitrogen, Carlsbad, CA, USA) with additional restriction enzyme cloning sites. PCR with the MCMV Smith DNA as the template was applied to produce the DNA sequence encoding the M43 open reading frame with 5′ primer 5M43 (5′-GGGAATTCCATATGGGTACCACAACGACGTTGACGGGGC-3′) and 3′ primer 3M43 (5′-CCGGAATTCGGTACCTTATTGATGTCGGCAACACAC-3′).

### 2.2. Salmonella Strains and Attenuated Salmonella Vaccines

*Salmonella typhimurium* clinical isolate ST14028s has been previously described [40,41]. *Salmonella typhimurium* aroA strain SL7207 was a gift from Dr. Bruce A. D. Stocker (Stanford University, Stanford, CA, USA) [42]. We employed the λ Red recombinase-based mutagenesis method [43] to create *Salmonella* strain ST625 from SL7207 by deleting a part of spiR. Following the previous described protocols [40,41], we created the spiR deletion mutagenesis cassette products by PCR with pKan-clone7 as the template. The created DNA products were introduced into SL7207. The nonpolar strain ST625 was isolated, and its spiR mutation was confirmed by sequencing analysis [40,41]. Functional vaccine Sal-M43 and control vaccine Sal-C were created by carrying transformation of ST625 with M43 expression construct pEXP-M43 and empty vector construct pEXP, respectively. We followed the previously reported protocols to conduct in vitro growth of various *Salmonella* strains in LB broth [40,41]. Each assay was performed in duplicate and repeated independently three times.

In experiments detecting M43, J774 macrophages were incubated with IFN-γ (150 U/mL) (R&D Systems Inc., Minneapolis, MN, USA), infected with Sal-M43 and Sal-C (MOI = 0.2), and collected at day 3 postinfection. Proteins were separated in SDS-containing polyacrylamide gels, electrically transferred, and stained with anti-M43 antisera manufactured by rabbits injected with M43 peptides (Promab, Inc., Richmond, CA, USA) and analyzed using a STORM840 Phosphorimager [40,44].

### 2.3. Animal Vaccination and Treatment

Four-week-old BALB/c mice (Jackson Laboratory, Bar Harbor, ME, USA) were first anesthetized and then intragastrically administered phosphate-buffered saline (PBS) without *Salmonella* or with 1 × 10^9^ cfu Sal-M43 or Sal-C [40,41]. We vaccinated the animals on days 0, 14, and 28 and carried out two trials with 5 mice per group for each experiment.

### 2.4. IgG and IgA ELISA and T Cell ELISPOT Assays

We gathered serum/mucosal wash samples following described procedures [45,46]. Infected NIH3T3 cell lysates with mutant m-M43 or Smith strain (MOI = 1) were produced at 96 h postinfection [31,37]. We applied ELISA assays for assessing the serum IgG and mucosal IgA activity to interact with the Smith-infected or m-M43-infected cell lysates. We added to the Medisorp plates (Thermo Fisher, Waltham, MA, USA) the MCMV-infected cell lysates and the serum or nasal wash samples and allowed them to react first with goat anti-mouse IgG AP or IgA AP secondary antibodies (Cell Signaling Technologies, Danvers, MA, USA). The samples were then stained chemiluminescently with reagents from BioLegend (San Diego, CA, USA) and read and evaluated with a Spectramax instrument (San Jose, CA, USA).

For assaying the number of IFN-γ-expressing T cells, IFN-γ ELISPOT kits (U-Cytech biosciences, Utrecht, The Netherlands) were used [47]. ELISPOT plates with splenocytes (1 × 10^6^ cells) were first incubated with MCMV-infected cell lysates, then stained with an IFN-γ anti-antibody, and examined [47]. Each assay was performed in duplicate and repeated independently three times.

### 2.5. Studies of MCMV Infection in Immunized Animals

Groups of BALB/c mice (Jackson Laboratory, Bar Harbor, ME, USA) (5–10 animals per group) were intranasally or intraperitoneally infected with salivary gland-passaged MCMV Smith strain (1 × 10^6^ PFU per mice) (for lethal dosage challenge) or cultured cell-passaged MCMV Smith strain (5 × 10^4^ PFU per mice) two weeks after the final immunization [31,37]. To prepare the salivary gland-passaged MCMV stocks, we infected BALB/c mice intraperitoneally with MCMV (Smith) (5 × 10^2^ PFU per mice) and harvested the salivary glands at 14 days postinfection. The harvested tissues were homogenized (10% wt/vol) in Dulbecco’s modified Eagle’s medium (DMEM) and subjected to centrifugation [31,37]. The resulting supernatants were aliquoted and stored in liquid nitrogen. We observed the mice with the lethal MCMV challenge dosage daily to record their survival for two weeks. Humane (non-lethal) endpoints were used during the survival experiments. Animals were deemed gravely ill and euthanized immediately if they exhibited lethargy, ruffled hair coat, hunched posture, or other similar signs of significant discomfort.

Salivary glands, livers, lungs, and spleens were isolated from mice challenged with cultured cell-passaged MCMV at day 5 postinfection [31,37]. We determined the viral titers in these organs using plaque assays in NIH 3T3 cells following the protocols as previously described [31,37]. Each assay was performed in duplicate and repeated independently three times.

### 2.6. Statistical Analysis

Each assay was performed in duplicate and repeated independently three times. All data are given as mean values and standard deviations (SDs). Statistical analyses using GraphPad Prism software (version 10) were conducted, and a *p*-value of less than 0.05 was viewed as significant.

### 2.7. Ethics Statement

All experiments were conducted following the recommendations of the *Guide for the Care and Use of Laboratory Animals* of the National Research Council. Efforts were applied to reduce animal suffering during experimental procedures. We monitored the animals daily, and humane (non-lethal) endpoints were used during the survival experiments. Animals were deemed gravely ill and euthanized immediately if they exhibited lethargy, ruffled hair coat, hunched posture, or other similar signs of significant discomfort. During euthanasia, carbon dioxide was delivered from a pressurized tank into a cage where the mice were placed. Mice were monitored for cessation of respiration and remained in the chamber for an additional 60 s after respiration had ceased. Mice were removed from the cage, and after confirming a lack of a palpable heartbeat, were subjected to cervical dislocation. The Animal Care and Use Committee (UC-Berkeley) approved the animal experiment protocol (Protocol #R240), including the euthanasia procedure, on 7 December 2022.

## 3. Results

### 3.1. Anti-CMV Vaccines Derived from Attenuated Salmonella

Our previous studies showed that the attenuated *Salmonella typhimurium* strain SL7207 [42] can deliver plasmid constructs containing gene-targeting RNase P ribozymes for efficient expression [14,44]. In this report, we deleted the SpiR gene sequence from *Salmonella typhimurium* strain SL7207 to create a new attenuated *Salmonella* strain, ST625. The SpiR protein functions as a master regulator that is required for the expression of many members of the type III secretion system (T3SS) of *Salmonella* pathogenicity island 2 (SPI-2) [25,26]. These members are major bacterial virulence factors that contribute significantly to *Salmonella* survival and replication in the infected cells [16,17]. *Salmonella* strains with the disruption of the expression of these genes are expected to exhibit reduced virulence and cytotoxicity, improved lysis, and increased gene transfer [14,18]. Thus, the constructed attenuated *Salmonella* strain, ST625, due to the spiR deletion, should have little virulence and possess an excellent gene transfer capability.

In this report, two vaccines were used and derived from ST625. The functional vaccine, Sal-M43, was created by transforming ST625 with plasmid pEXP-M43, which had the M43-expressing cassette with the coding sequence of the MCMV M43 open reading frame driven by a eukaryotic-expressing promoter. A negative control vaccine, Sal-C, was also created by transforming ST625 with the empty plasmid vector in the absence of any MCMV sequences.

### 3.2. The Growth and Gene Delivery Ability of the Vaccines

**Figure 1:**
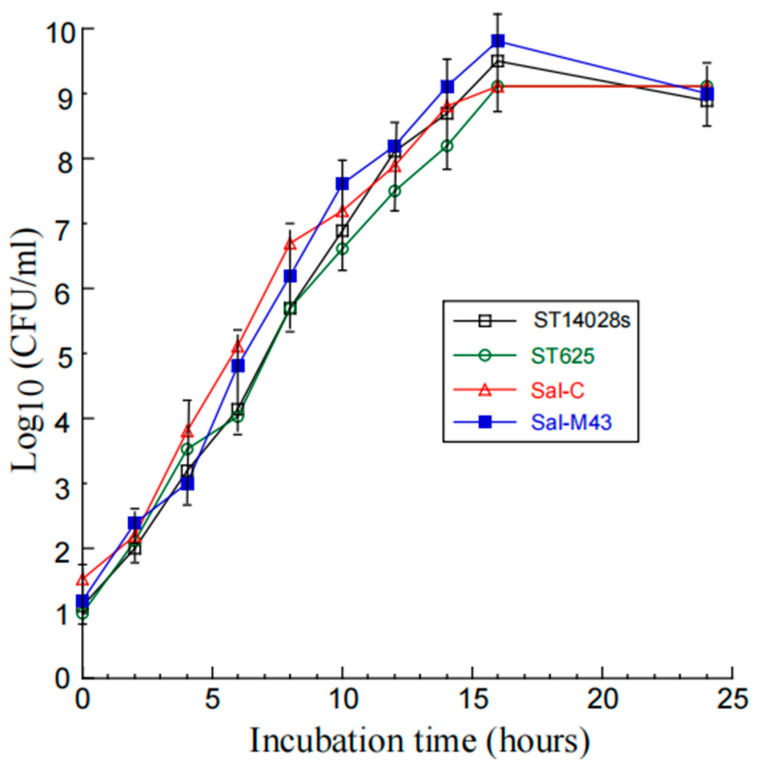
*Salmonella* growth in LB broth. We described the procedures of assaying the bacterial growth in Materials and Methods.

We executed three sets of experiments to investigate the growth and gene delivery ability of the constructed *Salmonella*-based vaccines. First, we observed no substantial difference in the in vitro growth (in LB broth) of the functional vaccine Sal-M43, the control vaccine Sal-C, the parental attenuated strain ST625 without any plasmid constructs, and a clinical strain ST14028s (Figure 1). Our findings imply that the presence of the vector construct and M43 expression cassette does not affect the growth and viability of the attenuated *Salmonella*.

**Figure 2:**
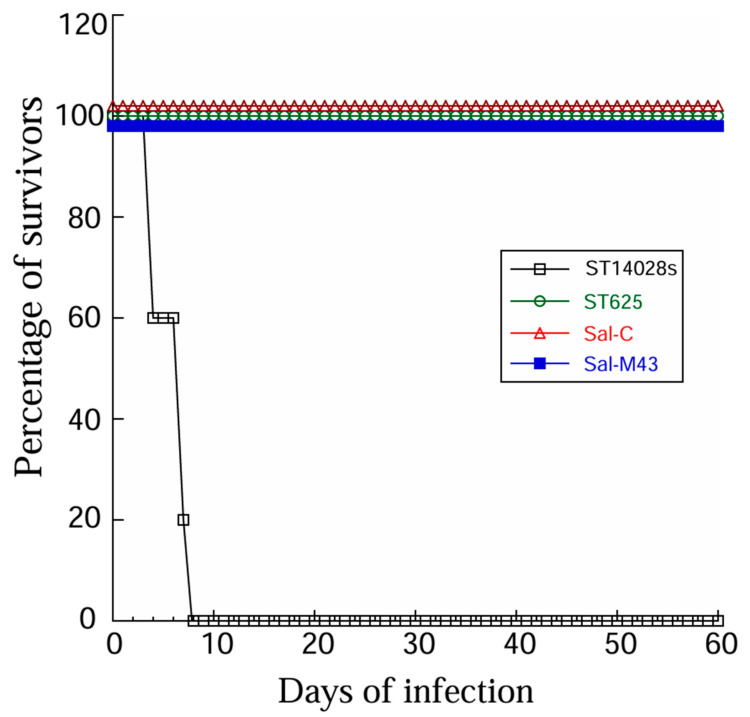
Survival of *Salmonella*-infected animals. We intragastrically infected mice (10 animals per group) with clinical strain ST14028s (2 × 10^3^ CFU) and ST625 (1 × 10^9^ CFU) and its derived vaccines Sal-C and Sal-M43.

Second, experiments in which mice were infected with these bacteria showed that the functional vaccine Sal-M43, the control vaccine Sal-C, and the parental strain ST625 were substantially attenuated in their capability for killing the animals, compared to the clinical strain ST14028s. We observed no death in animals infected with ST625, Sal-M43, and Sal-C (1 × 10^9^ cfu/mouse), even at 60 days postinfection (Figure 2), while no survival was found in animals infected by ST14028s with a lower dose inoculum (2 × 10^3^ cfu/mouse) beyond 7 days.

Third, the expression of the M43 protein (~70 KD) was detected in mouse J774 macrophages infected with the functional vaccine Sal-M43, which contained the M43 expression cassette, but not in cells infected with the control vaccine Sal-C, which contained only the empty vector (Supplementary Materials, Figure S1). The M43 protein was not detected when Sal-M43 was cultured alone in the LB broth without J774 macrophages, consistent with the observations that the M43 expression was driven by a eukaryotic expression promoter, which only turned on in mammalian cells. Thus, Sal-M43 is capable of completing the gene transfer for the expression of the M43 protein in mouse cells.

### 3.3. T Cell and Antibody Responses Induced by the Constructed Vaccines

**Figure 3:**
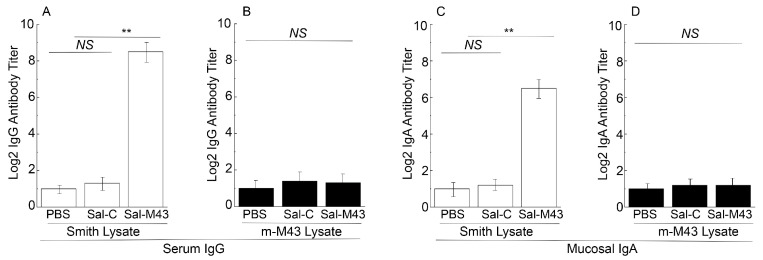
Serum IgG (**A**,**B**) and mucosal IgA titers (**C**,**D**) in immunized animals at 42 days postimmunization. Mice were intragastrically administered with PBS, Sal-C, and Sal-M43 at day 0, 14, and 28. ELISA assays were conducted with pool sera or mucosal washes using the infected lysates with Smith (**A**,**C**) or m-M43 (**B**,**D**). Each assay was performed in duplicate and repeated independently three times. ** *p* < 0.05. NS, not significant.

We inoculated mice intragastrically with Sal-M43, Sal-C, or phosphate-buffered saline (PBS)(as a negative control) at days 0, 14, and 28. At 42 days after immunization, the ELISA was applied for the determination of the levels of serum antibodies in mice (Figure 3). To study the functionality of the humoral responses from the vaccinated mice, we used two sets of lysates as the antigens in the ELISA experiments: the lysates of cells infected with the MCMV mutant m-M43 and those with the wildtype Smith strain. We created mutant m-M43 by deleting the M43 sequence from the Smith strain. MCMV mutants with mutations at M43 replicated well in some cell lines but were attenuated in virus production in other cell lines in vitro [30,31]. We have previously shown that an MCMV mutant with a transposon mutation at M43 is defective in growth in salivary glands in infected mice and M43 functions to modulate the T helper cell response [31,32]. We used the lysates of m-M43-infected cells to investigate if the antibody responses elicited from the mice immunized by the *Salmonella*-based vaccines were anti-M43-specific because the m-M43 virus lacked the M43 sequence and failed to manufacture M43.

In the ELISA experiments with the lysates of Smith strain-infected cells, anti-MCMV serum antibody titers in mice immunized with the functional vaccine Sal-M43 were at least 200-fold higher than those in mice with the control vaccine Sal-C (Figure 3A). In the experiments with the lysates of cells infected with mutant m-M43 without the M43 expression, however, we barely detected anti-MCMV serum antibody titers from the Sal-M43- and Sal-C immunized mice (Figure 3B). Thus, the serum humoral responses induced by the Sal-M43 immunization appear to be specific for M43.

*Salmonella* and its attenuated strains are known to infect and colonize in the gastrointestinal tract and stimulate mucosal immune responses [12]. To investigate if the *Salmonella*-based vaccines induce mucosal antibody responses, nasal washes were collected from the mice at 0, 16, 32, and 42 days after immunization and assayed for anti-MCMV IgA levels. In the ELISA experiments with the lysates of the Smith strain-infected cells, we observed an at least 50-fold increase in anti-MCMV IgA antibody titers in mice immunized with the functional vaccine Sal-M43 at 42 days postimmunization, compared to those with the control vaccine Sal-C (Figure 3C). In the experiments with the lysates of cells infected with the mutant m-M43 without the M43 expression, however, we barely detected anti-MCMV IgA antibody titers from the Sal-M43- and Sal-C-immunized mice (Figure 3D). Thus, the mucosal IgA responses induced by the Sal-M43 immunization appeared to be specific for M43.

**Figure 4:**
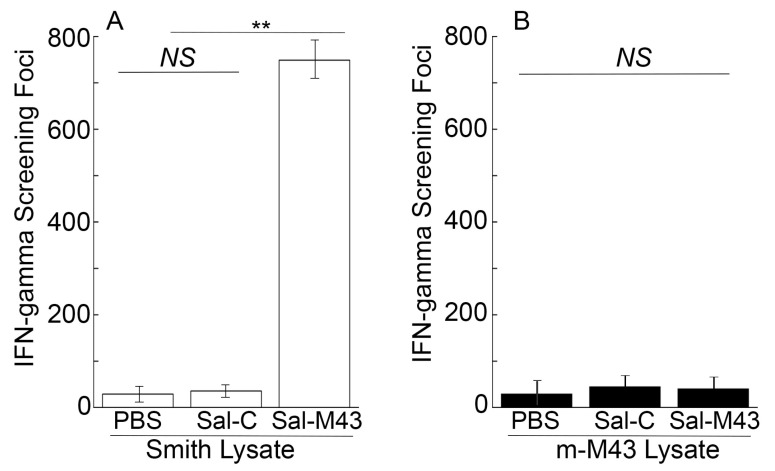
Levels of IFN-γ-producing T cells from splenocytes in immunized mice at 42 days postimmunization. Mice were intragastrically administered with PBS, Sal-C, and Sal-M43 at day 0, 14, and 28. ELISPOT assays were conducted with pool splenocytes using the infected lysates with Smith (**A**) or m-M43 (**B**), with the results presented as spot-forming cells (SFCs) per million cells. Each assay was performed in duplicate and repeated independently three times. ** *p* < 0.05. NS, not significant.

To study the T cell responses induced by the constructed vaccines, splenocytes were collected 42 days after the oral administration and incubated with the lysates of Smith-infected and m-M43-infeced cells. In the ELISPOT experiments with the lysates of Smith strain-infected cells, we observed at least a 70-fold increase in anti-MCMV IFN-γ-producing T cell responses in Sal-M43-vaccinated mice compared to Sal-C-vaccinated mice (Figure 4). In the experiments with the lysates of cells infected with mutant m-M43 without the M43 expression, however, we barely detected anti-MCMV T cell responses from the Sal-M43- and Sal-C-immunized mice (Figure 4). Thus, Sal-M43 also induced anti-MCMV T cell responses that were specific for M43.

### 3.4. Immune Protection of Mice from MCMV Challenge by the Constructed Vaccine

**Figure 5:**
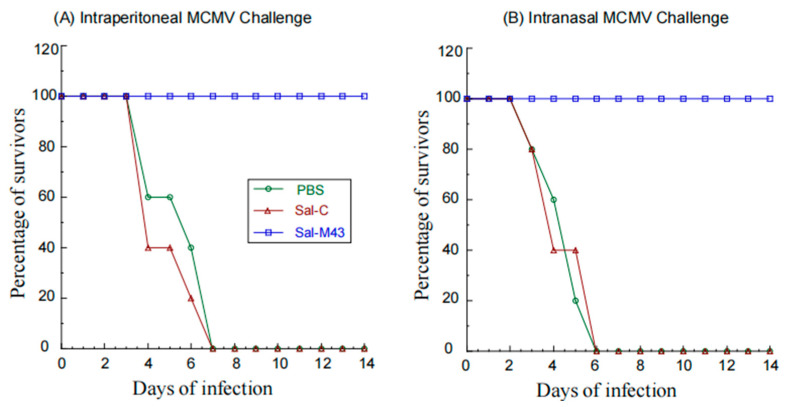
Survival of immunized mice after MCMV challenge. We intragastrically administered mice (10 mice per group) at day 0, 14, and 28 with PBS, Sal-C, and Sal-M43 and then challenged the animals intraperitoneally (**A**) or intranasally (**B**) with MCMV Smith (1 × 10^6^ PFU) at two weeks after the final immunization. The percentage of survival is shown.

Two series of experiments were conducted to investigate if the vaccine with the expression of M43 could induce antiviral immunity against the systemic and mucosal MCMV challenge, respectively. Animals were administered with PBS, Sal-C, and Sal-M43 at days 0, 14, and 28 and then either intranasally or intraperitoneally challenged with a lethal dose of salivary gland-passaged highly pathogenic MCMV at day 42. No survival was found in mice with the PBS or control vaccine Sal-C beyond 7 days postinfection (Figure 5). In contrast, we found no death in mice that were immunized with the functional vaccine Sal-M43, even at 14 days postinfection (Figure 5). Thus, the ST625-M43 immunization provides 100% antiviral immunity against the systemic and mucosal MCMV lethal challenge.

### 3.5. Inhibition of MCMV Infection in Mice Immunized with Salmonella-Based Vaccine

**Figure 6:**
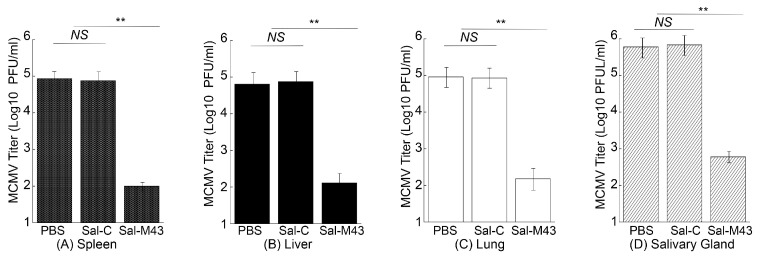
Reduced virus growth in Sal-M43-immunized mice intraperitoneally challenged with MCMV. We intragastrically administered mice (10 mice per group) at day 0, 14, and 28 with PBS, Sal-C, and Sal-M43 and intraperitoneally inoculated the animals with Smith (5 × 10^4^ PFU) at two weeks after the final vaccination. We assessed the amounts of virus in the spleens (**A**), livers (**B**), lungs (**C**), and salivary glands (**D**) of the animals at 120 h after MCMV infection. Each assay was performed in duplicate and repeated independently three times. ** *p* < 0.05. NS, not significant.

**Figure 7:**
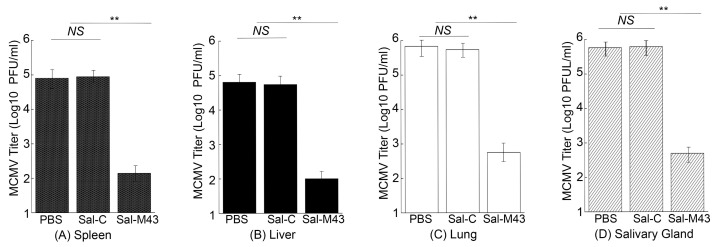
Reduced virus growth in Sal-M43-immunized mice intranasally challenged with MCMV. We intragastrically administered mice (10 mice per group) at day 0, 14, and 28 with PBS, Sal-C, and Sal-M43 and intranasally inoculated the animals with Smith (5 × 10^4^ PFU) at two weeks after the final vaccination. We assessed the amounts of virus in the spleens (**A**), livers (**B**), lungs (**C**), and salivary glands (**D**) of the animals at 120 h after MCMV infection. Each assay was performed in duplicate and repeated independently three times. ** *p* < 0.05. NS, not significant.

To examine the MCMV infection in immunized mice, we administered mice with PBS, Sal-C, and Sal-M43 at day 0, 14, and 28 and then either intraperitoneally or intranasally challenged these animals with a sub-lethal dose of MCMV at day 42. At day 5 post-challenge, we collected various organs and measured MCMV titers in these organs with a plaque assay. In the mice intraperitoneally challenged with MCMV, the amounts of the virus in the salivary glands, lungs, livers, and spleens in animals immunized with the functional vaccine Sal-M43 were approximately 1000-, 600-, 500-, and 850-fold less than those in the PBS-treated mice, respectively (Figure 6). On the contrary, the amounts of the virus in the Sal-C-vaccinated mice were found to be comparable to those in PBS-treated animals (Figure 6A–D). When intranasally challenged with MCMV, the vaccinated mice also exhibited similar results (Figure 7). In these experiments, the amounts of the virus in the salivary glands, lungs, livers, and spleens in animals immunized with Sal-M43 were about 1200-, 1200-, 650-, and 650-fold less than those in PBS-treated mice, respectively. Contrariwise, the amounts of the virus in these tissues in Sal-C-vaccinated mice were comparable to those in PBS-treated animals (Figure 7A–D). Thus, the Sal-M43 vaccination inhibits the MCMV infection and growth in the intranasally and intraperitoneally challenged mice.

## 4. Discussion

HCMV is a medically important virus with global public health consequences [1,7]. This virus causes substantial morbidity and mortality in neonates and immunosuppressed people [1]. Moreover, HCMV is ubiquitous and causes a substantial economic burden to society [5,6]. Generating an anti-HCMV vaccine is required for preventing the infection and associated diseases of human CMV [8,9].

Oral vaccines against HCMV are attractive since these vaccines are inexpensive and can be applied orally and easily for mass immunization. Attenuated *Salmonella* was previously investigated in our laboratory as a gene transfer vector to deliver the expression cassettes of antiviral ribozymes in cultured cells [44]. In this study, we created a new attenuated *Salmonella typhimurium* strain, ST625, as an oral gene transfer vector for the expression of the MCMV M43 open reading frame. ST625 contained a deletion of the spiR gene, which encodes a master regulator protein required for the expression of many SPI-2 T3SS factors [25,26]. We showed that mice immunized with our constructed functional *Salmonella*-based vaccine, Sal-M43, exhibited an elevated anti-MCMV immunity specifically against the M43 antigen. Moreover, these Sal-M43-immunized animals were completely protected from the mucosal and systemic MCMV lethal challenge. Thus, our study provides the first direct evidence that M43-expressing vaccines based on attenuated *Salmonella* (spiR-) strains can be used as oral vaccine candidates against MCMV infections in mice.

Attenuated *Salmonella* has been used against typhoid fever [48,49] and can be engineered as a gene transfer vector for oral vaccinations against pathogens [11,12]. However, several issues need to be addressed to develop attenuated *Salmonella*-based vaccines against CMV infections. First, further studies on the “pharmacokinetic” properties of bacteria in vivo, such as the *Salmonella* turnover/clearance after vaccination, should be performed to eliminate potential safety concerns associated with live bacteria. Second, the identification of bacterial virulent factors should lead to the manufacture of new *Salmonella* vectors disabling the factors, which may exhibit reduced virulence and pathogenicity and increased safety profiles in vivo. Third, the investigation of the intracellular gene transfer mechanism of *Salmonella*-based vectors should facilitate the development of novel strains with increased gene delivery efficiency. Our constructed *Salmonella* strain, ST625, contained a deletion at spiR, which functions as a master regulator required for the production of many SPI-2 T3SS components [25,26]. These components are major bacterial virulence factors that contribute significantly to *Salmonella* survival and replication in the infected cells [16,17]. The disruption of the expression of these genes should lead to reduced virulence and cytotoxicity, improved lysis, and increased gene transfer ability of the mutated *Salmonella* strains [14,18]. Indeed, ST625, with the spiR deletion, showed little virulence/pathogenicity and an excellent gene transfer ability (Figure 1 and Figure 2). Thus, ST625 represents a promising gene transfer vector for gene delivery applications.

Anti-CMV vaccine studies in animals and humans have been conducted with numerous designs of candidates, such as recombinant protein and DNA vaccines, virus-like particle (VLP) vaccines, and conventional inactivated whole viral vaccines [8]. Some of these vaccine investigations yielded promising results, such as vaccine-induced effective humoral and T cell immune responses and immune protection from MCMV challenges [20,21,22,23,24]. It is not known if M43 or its HCMV homolog, UL43, can be an antigen for anti-CMV vaccine development. M43 is not essential for MCMV propagation in vitro, as viral mutants inactivating M43 grew well in several cell lines but not other cell lines [30,31]. Our previous results also showed that M43 functions to modulate the T helper cell response, and an MCMV mutant with a transposon mutation at M43 was defective in growth in salivary glands in infected mice [31,32]. Like M43, UL43 is also dispensable for HCMV propagation in vitro [33,34]. Working with HCMV UL24 together, UL43 functions to modulate the SAMHD1 protein cellular distribution and UL16 binding protein 1 production [35,36].

The results presented in this report revealed that Sal-M43 induced substantial anti-MCMV immunity in immunized mice. Moreover, the constructed vaccine offered 100% protection from the lethal viral challenge in immunized mice. Little is currently known about if the induced antibody responses in our study can neutralize the infectivity of virus infectious particles, which contain tegument protein M43, while it is still not known if anti-UL43 antibodies generated previously exhibit neutralizing activity against HCMV infectious virions, which contain UL43, a tegument protein [27,28,29]. The role of M43/UL43 as a tegument protein does not necessarily influence its ability to act as a T-cell target, as it can just be expressed in target cells or taken up by antigen-presenting cells. It is noted in our previous studies that M43 functions to modulate the T helper cell response, and an MCMV mutant with a transposon mutation at M43 was defective in growth in salivary glands in infected mice [31,32]. Additional experiments can be performed to investigate these important topics and understand the capacity of M43 to stimulate antiviral immunity and the potential of the induced immunity against viruses and their infectivity.

Our current study demonstrates the potential of developing Sal-M43 as an anti-CMV oral vaccine. Previous investigations in animals [20,21,22,23,24] and humans [8] have been performed to evaluate anti-CMV vaccines expressing different viral proteins (e.g., HCMV pentamer, gB, IE1, UL83, and their MCMV counterparts) and using different vaccine designs (e.g., recombinant protein and DNA vaccines, inactivated viral antigen and attenuated viral strain vaccines). Our current study focused on M43 as the target antigen. It will be interesting to construct ST625-based vaccines with the expression of M43/UL43 and other viral antigens, such as HCMV pentamer, gB, IE1, and their MCMV counterparts, and evaluate the ability of these vaccines in inducing antiviral immune responses. Additional vaccine studies of *Salmonella*-based vectors, such as ST625, for the expression of different CMV antigens in animals and humans should facilitate our efforts in developing an anti-CMV vaccine to block viral transmission and prevent virus-associated diseases.

## Data Availability

The dataset is available on request from the authors.

## Supplementary Materials

The following supporting information can be downloaded at https://www.mdpi.com/article/10.3390/pathogens14090902/s1: Figure S1: M43 expression detected by Western blot experiments.
