# Supplementary figures and images for: An Oral Salmonella-Based Vaccine Expressing Viral M43 Protein Elicits Effective Immunity Against Murine Cytomegalovirus in Mice
Source: Pathogens. 2025 Sep 8;14(9):902. doi: 10.3390/pathogens14090902 (PMC12472285; doi:10.3390/pathogens14090902)

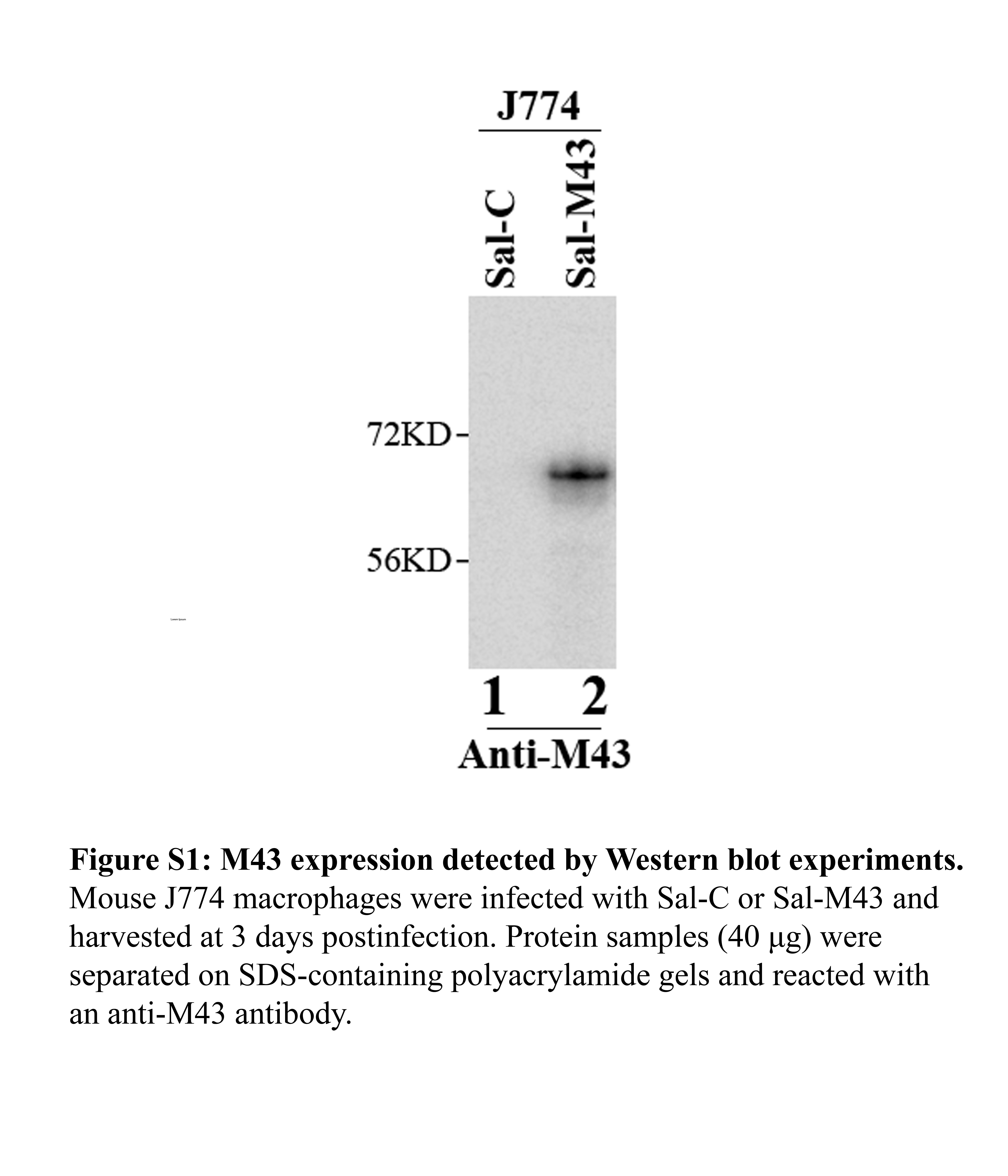

Supplement: Supplementary file 1 [file pathogens-14-00902-s001.zip › pathogens-3751073-supplementary.jpg]
